# Supplementary material for: I’m fine with collecting data: Engagement profiles differ depending on scientific activities in an online community of a citizen science project
Source: PLoS One. 2022 Oct 10;17(10):e0275785. doi: 10.1371/journal.pone.0275785 (PMC9551629; doi:10.1371/journal.pone.0275785)

Supporting information file 2

Fig S2. Within-groups sums of squares for clustering of 2 to 10 groups (clusters) for (1) the whole project 'Wildlife Researchers 1' (*N* = 131), (2) the data collection phase (*N* = 121) and (3) the data analysis phase (*N* = 36).

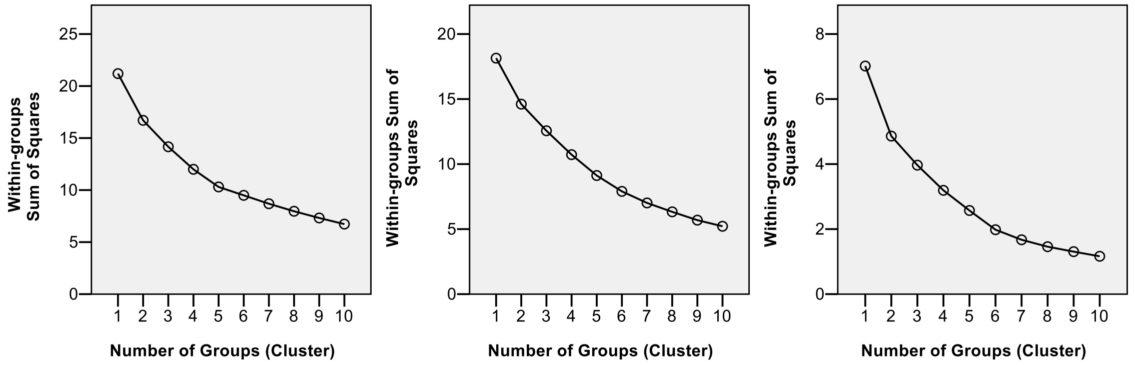

Supplement: S2 File — (PDF) [file pone.0275785.s002.pdf]
