# Supplementary material for: I’m fine with collecting data: Engagement profiles differ depending on scientific activities in an online community of a citizen science project
Source: PLoS One. 2022 Oct 10;17(10):e0275785. doi: 10.1371/journal.pone.0275785 (PMC9551629; doi:10.1371/journal.pone.0275785)
Supplement: S3 File — (PDF) [file pone.0275785.s003.pdf]

Supporting information file 3

To generate an interactive version of the Sankey diagram please Copy-Paste the following code sequence on the Load > Your Own Data -Option on the following page: http://sankey-diagram-generator.acquireprocure.com/ [Accessed on the 24.03.2022]

{"nodes":[{"name":" loyal","node":0,"color":"#cccccc"},{"name":" hardworking","node":1,"color":"#cccccc"},{"name":" lurker","node":2,"color":"#cccccc"},{"name":" moderate","node":3,"color":"#cccccc"},{"name":" contributor","node":4,"color":"#cccccc"},{"name":" visitor","node":5,"color":"#cccccc"},{"name":" persistent","node":6,"color":"#cccccc"},{"name":"visitor","node":7,"color":"#cccccc","xPos":1166,"yPos":0},{"name":"loyal","node":8,"color":"#cccccc","xPos":1166,"yPos":382},{"name":"moderate","node":9,"color":"#cccccc","xPos":1166,"yPos":321},{"name":"lasting","node":10,"color":"#cccccc","xPos":1166,"yPos":435},{"name":"persistent","node":11,"color":"#cccccc","xPos":1166,"yPos":525},{"name":"lurker","node":12,"color":"#cccccc","xPos":1166,"yPos":483},{"name":"hardworking","node":13,"color":"#cccccc","xPos":1166,"yPos":558}],"links":[{"id":0,"value":41,"source":0,"target":7,"color":"#666666"},{"id":8,"value":3,"source":0,"target":8},{"id":6,"value":5,"source":0,"target":9},{"id":9,"value":3,"source":0,"target":10},{"id":10,"value":3,"source":1,"target":7},{"id":16,"value":1,"source":1,"target":9},{"id":3,"value":10,"source":2,"target":7,"color":"#666666"},{"id":17,"value":1,"source":2,"target":8,"color":"#cccccc"},{"id":14,"value":2,"source":2,"target":9,"color":"#cccccc"},{"id":15,"value":2,"source":2,"target":12},{"id":4,"value":8,"source":3,"target":7,"color":"#666666"},{"id":1,"value":20,"source":4,"target":7,"color":"#666666"},{"id":7,"value":5,"source":4,"target":8,"color":"#cccccc"},{"id":11,"value":3,"source":4,"target":9},{"id":12,"value":3,"source":4,"target":10},{"id":18,"value":1,"source":4,"target":11},{"id":13,"value":3,"source":4,"t

arget":12},{"id":2,"value":17,"source":5,"target":7},{"id":19,"value":1,"source":5,"target":9},{"id":20,"value":1,"source":5,"target":10},{"id":21,"value":1,"source":5,"target":13},{"id":5,"value":6,"source":6,"target":7,"color":"#666666"},{"id":22,"value":1,"source":6,"target":11}],"layout":"hyrachical","opacity":"0.3","density":"28"}
